# Characteristics, management, and in-hospital mortality among patients with severe sepsis in intensive care units in Japan: the FORECAST study

**DOI:** 10.1186/s13054-018-2186-7

**Published:** 2018-11-22

**Authors:** Toshikazu Abe, Hiroshi Ogura, Atsushi Shiraishi, Shigeki Kushimoto, Daizoh Saitoh, Seitaro Fujishima, Toshihiko Mayumi, Yasukazu Shiino, Taka-aki Nakada, Takehiko Tarui, Toru Hifumi, Yasuhiro Otomo, Kohji Okamoto, Yutaka Umemura, Joji Kotani, Yuichiro Sakamoto, Junichi Sasaki, Shin-ichiro Shiraishi, Kiyotsugu Takuma, Ryosuke Tsuruta, Akiyoshi Hagiwara, Kazuma Yamakawa, Tomohiko Masuno, Naoshi Takeyama, Norio Yamashita, Hiroto Ikeda, Masashi Ueyama, Satoshi Fujimi, Satoshi Gando, on behalf of JAAM FORECAST group, Osamu Tasaki, Yasumitsu Mizobata, Hiraku Funakoshi, Toshiro Okuyama, Iwao Yamashita, Toshio Kanai, Yasuo Yamada, Mayuki Aibiki, Keiji Sato, Susumu Yamashita, Kenichi Yoshida, Shunji Kasaoka, Akihide Kon, Hiroshi Rinka, Hiroshi Kato, Hiroshi Okudera, Eichi Narimatsu, Toshifumi Fujiwara, Manabu Sugita, Yasuo Shichinohe, Hajime Nakae, Ryouji Iiduka, Mitsunobu Nakamura, Yuji Murata, Yoshitake Sato, Hiroyasu Ishikura, Yasuhiro Myojo, Yasuyuki Tsujita, Kosaku Kinoshita, Hiroyuki Yamaguchi, Toshihiro Sakurai, Satoru Miyatake, Takao Saotome, Susumu Yasuda, Toshikazu Abe, Hiroshi Ogura, Yutaka Umemura, Atsushi Shiraishi, Shigeki Kushimoto, Daizoh Saitoh, Seitaro Fujishima, Junichi Sasaki, Toshihiko Mayumi, Yasukazu Shiino, Taka-aki Nakada, Takehiko Tarui, Toru Hifumi, Yasuhiro Otomo, Joji Kotani, Yuichiro Sakamoto, Shin-ichiro Shiraishi, Kiyotsugu Takuma, Ryosuke Tsuruta, Akiyoshi Hagiwara, Kazuma Yamakawa, Naoshi Takeyama, Norio Yamashita, Hiroto Ikeda, Yasuaki Mizushima, Satoshi Gando

**Affiliations:** 1 Department of General Medicine, Juntendo University, 2-1-1, Hongo, Bunkyo-ku, Tokyo, 113-0033 Japan; 2 Health Services Research and Development Center, University of Tsukuba, Tsukuba, Japan; 3 Department of Traumatology and Acute Critical Medicine, Osaka University Graduate School of Medicine, Osaka, Japan; 4 Emergency and Trauma Center, Kameda Medical Center, Kamogawa, Japan; 5 Division of Emergency and Critical Care Medicine, Tohoku University Graduate School of Medicine, Sendai, Japan; 6 Division of Traumatology, Research Institute, National Defense Medical College, Tokorozawa, Japan; 7 Center for General Medicine Education, Keio University School of Medicine, Tokyo, Japan; 8 Department of Emergency Medicine, School of Medicine, University of Occupational and Environmental Health, Kitakyushu, Japan; 9 Department of Acute Medicine, Kawasaki Medical School, Kurashiki, Japan; 10 Department of Emergency and Critical Care Medicine Chiba University Graduate School of Medicine, Chiba, Japan; 11 Department of Trauma and Critical Care Medicine, Kyorin University School of Medicine, Tokyo, Japan; 12 Department of Emergency and Critical Care Medicine, St. Luke’s International Hospital, Tokyo, Japan; 13 Trauma and Acute Critical Care Center, Medical Hospital, Tokyo Medical and Dental University, Tokyo, Japan; 14 Department of Surgery, Center for Gastroenterology and Liver Disease, Kitakyushu City Yahata Hospital, Kitakyushu, Japan; 15 Department of Disaster and Emergency Medicine, Kobe University Graduate School of Medicine, Kobe, Japan; 16 Emergency and Critical Care Medicine, Saga University Hospital, Saga, Japan; 17 Department of Emergency and Critical Care Medicine, Keio University School of Medicine, Tokyo, Japan; 18 Department of Emergency and Critical Care Medicine, Aizu Chuo Hospital, Aizuwakamatsu, Japan; 19 Emergency & Critical Care Center, Kawasaki Municipal Kawasaki Hospital, Kawasaki, Japan; 20 Advanced Medical Emergency & Critical Care Center, Yamaguchi University Hospital, Ube, Japan; 21 Center Hospital of the National Center for Global Health and Medicine, Tokyo, Japan; 22 Division of Trauma and Surgical Critical Care, Osaka General Medical Center, Osaka, Japan; 23 Department of Emergency and Critical Care Medicine, Nippon Medical School, Tokyo, Japan; 24 Advanced Critical Care Center, Aichi Medical University Hospital, Nagakute, Japan; 25 Advanced Emergency Medical Service Center Kurume University Hospital, Kurume, Japan; 26 Department of Emergency Medicine, Teikyo University School of Medicine, Tokyo, Japan; 27 Department of Trauma, Critical Care Medicine, and Burn Center, Japan Community Healthcare Organization, Chukyo Hospital, Nagoya, Japan; 28 Division of Acute and Critical Care Medicine, Hokkaido University Graduate School of Medicine, Sapporo, Japan

**Keywords:** Sepsis, Bundle, Resuscitation, Elderly

## Abstract

**Background:**

Sepsis is a leading cause of death and long-term disability in developed countries. A comprehensive report on the incidence, clinical characteristics, and evolving management of sepsis is important. Thus, this study aimed to evaluate the characteristics, management, and outcomes of patients with severe sepsis in Japan.

**Methods:**

This is a cohort study of the Focused Outcomes Research in Emergency Care in Acute Respiratory Distress Syndrome, Sepsis, and Trauma (FORECAST) study, which was a multicenter, prospective cohort study conducted at 59 intensive care units (ICUs) from January 2016 to March 2017. We included adult patients with severe sepsis based on the sepsis-2 criteria.

**Results:**

In total, 1184 patients (median age 73 years, interquartile range (IQR) 64–81) with severe sepsis were admitted to the ICU during the study period. The most common comorbidity was diabetes mellitus (23%). Moreover, approximately 63% of patients had septic shock. The median Sepsis-related Organ Failure Assessment (SOFA) score was 9 (IQR 6–11). The most common site of infection was the lung (31%). Approximately 54% of the participants had positive blood cultures. The compliance rates for the entire 3-h bundle, measurement of central venous pressure, and assessment of central venous oxygen saturation were 64%, 26%, and 7%, respectively. A multilevel logistic regression model showed that closed ICUs and non-university hospitals were more compliant with the entire 3-h bundle. The in-hospital mortality rate of patients with severe sepsis was 23% (21–26%). Older age, multiple comorbidities, suspected site of infection, and increasing SOFA scores correlated with in-hospital mortality, based on the generalized estimating equation model. The length of hospital stay was 24 (12–46) days. Approximately 37% of the patients were discharged home after recovery.

**Conclusion:**

Our prospective study showed that sepsis management in Japan was characterized by a high compliance rate for the 3-h bundle and low compliance rate for central venous catheter measurements. The in-hospital mortality rate in Japan was comparable to that of other developed countries. Only one third of the patients were discharged home, considering the aging population with multiple comorbidities in the ICUs in Japan.

**Trial registration:**

UMIN-CTR, UMIN000019742. Registered on 16 November 2015.

**Electronic supplementary material:**

The online version of this article (10.1186/s13054-018-2186-7) contains supplementary material, which is available to authorized users.

## Background

Sepsis is a leading cause of admission to intensive care units (ICUs) and of death in developed countries [1, 2], and survivors have long-term disabilities. Several developed countries are undergoing demographic alterations due to aging, and changes in clinical practice and outcomes have not been sufficiently evaluated.

The Barcelona Declaration, which was promoted in the Surviving Sepsis Campaign (SSC) in 2002, aimed to reduce the mortality rate from sepsis by 25% within 5 years [3]. However, the impact of the campaign, including the utilization of care bundles and improving outcomes, remains limited despite ongoing revisions every 4 years. Relative to other high-profile diseases, such as cancer, sepsis has remained in the backwater despite a relatively long history of improvement in practice and research [4].

For optimal sepsis management, comprehensive treatment guidelines, such as those provided by the SSC, and information on local factors are essential. The Japanese Association for Acute Medicine Sepsis Registry study group investigated the epidemiology of severe sepsis in patients admitted to 15 ICUs in Japan in 2011, and their findings were reported in 2014 [5]. However, despite the fact that Japan is a developed and high-income country, larger, more comprehensive follow-up reports on the incidence, clinical characteristics, and evolving management of sepsis in Japan are not available. The World Health Assembly has recommended monitoring this progress, including the use of national registries, in an effort towards improving outcomes for both patients and survivors [4]. To increase awareness about sepsis and to reduce the burden of sepsis management, we established the Focused Outcomes Research in Emergency Care in Acute Respiratory Distress Syndrome, Sepsis, and Trauma (FORECAST) study to comprehensively describe the epidemiology and outcomes associated with severe sepsis and to understand how clinicians use guidelines in routine clinical practice in Japan.

## Methods

### Design and setting

A cohort of patients with severe sepsis was enrolled into the FORECAST study, which was a multicenter, prospective study on acutely ill patients, including those with acute respiratory distress syndrome, sepsis, and trauma. The FORECAST study used a consecutive sample of 59 ICUs in Japan, and it was conducted from January 2016 to March 2017. The FORECAST study was registered in the University Hospital Medical Information Network Clinical Trials Registry (UMIN-CTR ID: UMIN000019742).

### Participants

We included adult patients (age ≥ 16 years) with severe sepsis based on the sepsis-2 criteria published in 2003 [6]. All patients were admitted to the ICU. The inclusion criteria were as follows: patients suspected to have or was diagnosed with new-onset infection based on the history of the present illness; patients who met ≥ 2 systemic inflammatory response syndrome criteria; and patients who had at least one organ dysfunction. The sepsis-2 criteria also included the following: systolic blood pressure < 90 mmHg, mean arterial pressure (MAP) < 65 mmHg, or low blood pressure > 40 mmHg; serum creatinine > 2.0 mg/dL or diuresis (urine output < 0.5 mL/kg/h); total bilirubin > 2.0 mg/dL; platelet count < 100,000 cells/mm^3^; arterial lactate > 2 mmoL/L; international normalized ratio > 1.5; and arterial hypoxemia (partial pressure of arterial oxygen (PaO2)/fraction of inspired oxygen (FIO2) < 200) with pneumonia or PaO2/FIO2 < 250 without pneumonia) [6]. The exclusion criteria included the limitation of sustained life care or post-cardiopulmonary arrest resuscitation status at the time of sepsis diagnosis.

### Data collection

Data, which were compiled by FORECAST investigators, were obtained from the FORECAST database. Patient information included the demographic characteristics of the patients, admission source, various comorbidities, activities of daily living (ADL), suspected sites of infection, organ dysfunctions, sepsis-related severity scores, microbiology test results, and details of antibiotic use. In addition, we obtained data on compliance with established sepsis care protocols, such as the measurement of serum lactate within 3 h. Data collection was performed as part of the routine clinical workup. The primary outcome was in-hospital mortality. The secondary outcomes included status after discharge, ICU-free days, ventilator-free days (VFD), and length of hospital stay (LOS).

### Data definitions

Septic shock was defined based on the sepsis-2 criteria [6]. In individuals with organ dysfunction, hypotension was defined as systolic blood pressure < 90 mmHg, MAP < 65 mmHg, or decreasing blood pressure > 40 mmHg. Acute lung injury included the presence of arterial hypoxemia (PaO2/FIO2 < 200 with pneumonia or PaO2/FIO2 < 250 without pneumonia). Regarding comorbidities, cases of diabetes mellitus with and without end-organ complications were reported. Malignancies included solid, blood, and metastatic varieties, and cerebral vascular diseases included stroke and hemiplegia. Details are presented in Table 2. The Charlson comorbidity index (CCI) was classified into four previously defined grades: 0, none; 1–2, low; 3–4, moderate; and ≥ 5 points, high [7]. Based on the results of the blood culture tests, we excluded contamination if an investigator clinically confirmed contamination. We also measured compliance with the bundles proposed in the Surviving Sepsis Campaign Guidelines (SSCG) 2012 [8]. We defined compliance as evidence that all bundle elements were achieved within the appropriate time frame (i.e., 3 h or 6 h), and that they adhered to the indications (i.e., septic shock or lactate level > 4 mmol/L). In addition, VFD was defined as the number of days within the first 28 days after enrolment during which a patient was able to breathe without a ventilator. VFD in patients who died during the study period was assigned as 0. ICU-free days were calculated in the same manner.

### Analysis

Descriptive statistics included proportions for categorical variables, and the medians (interquartile range (IQR)) of the continuous variables were calculated because not all variables had normal distributions. There were few missing data (except bundle data); however, no assumptions were made about such data. Moreover, we analyzed the compliance rates for each bundle element and for the entire 3-h or 6-h bundles. Since data on the achievement of central venous pressure (CVP) of 8 mmHg and central venous oxygen saturation (ScvO2) of 70% in more than half of the participants were missing, we excluded these from the 6-h bundle analysis. To assess for compliance with the 2018 updated guidelines on the 1-h SSCG bundle of care [9], we calculated the compliance rates for the entire 3-h bundle in addition to vasopressor use to maintain MAP ≥ 65 mmHg and lactate re-measurement. To identify the factors correlated with compliance with the entire 3-h bundle considered for clustering by the ICUs, we developed a multilevel logistic regression model. Because bundle compliance usually correlates with institutional and service provider factors more than factors associated with the patients, we selected the following variables: type of ICU (open or closed), number of ICU beds, type of hospital (university hospital or other), number of board-certified intensivists, nurse-to-patient ratio, and number of ICU patients, after carefully examining clinically plausible interactions and multicollinearity. We did not evaluate the 6-h bundle because of several missing data.

Survival analysis using the Kaplan–Meier approach was performed to investigate the time from survival to discharge in patients with severe sepsis. The survival curve was drawn for 80 days, which showed that 88% of the patients were discharged. To identify the effect of the different epidemiological and treatment factors on in-hospital mortality considered for clustering by ICUs, we subsequently developed the generalized estimating equation (GEE) model with exchangeable working-correlation matrix. Age, sex, body mass index (BMI), ADL, admission source (emergency department (ED) or transferred from another hospital or ICU), CCI, presence of shock, suspected site of infection, Sepsis-related Organ Failure Assessment (SOFA) score, bacteremia, use of the fluid resuscitation protocol (the bundle in the SSCG 2012: administration of 30 mg/kg of crystalloid fluid bolus), and use of broad-spectrum antibiotics (the bundle in the SSCG 2012) were selected based on previous reports and clinical importance.

Statistical analyses were performed using the Statistical Package for Social Sciences software version 23.0 (IBM, Armonk, NY, USA) and Stata software version 15.1 (StataCorp, Texas, USA).

## Results

In total, 1184 patients with severe sepsis were included in the FORECAST study. Table 1 shows the organizational characteristics of the FORECAST study. It mainly consisted of the following: open ICU (61.0%), mixed ICU (94.9%), 11–20 ICU beds (44.1%), university hospitals (49.2%), and high-volume centers (78.0%; number of hospital beds ≥ 501). Table 2 presents the baseline characteristics of the participants. The median age of the participants was 73 years (IQR 64–81), and approximately 60.7% were men. The majority of the patients were admitted to the ICU directly from the ED (57.2%). Approximately 67.0% of the patients had at least one comorbidity. The most common comorbidity was diabetes mellitus (23.0%), followed by malignancies (17.7%), cerebrovascular diseases (11.8%), and congestive heart failure (10.8%). Approximately 24.3% of patients were deemed to be dependent as evaluated by ADL. The most common site of infection was the lung (31.0%), followed by the abdomen (26.3%), urinary tract (18.4%), and soft tissues (9.9%). Approximately 62.9% of patients had septic shock. Hypotension (55.4%) and hyperlactatemia (67.3%) were frequently observed in individuals with organ failure. The median Acute Physiologic Assessment and Chronic Health Evaluation II (APACHE II) score was 23 (IQR 17–29) and the SOFA score was 9 (IQR 6–11). Fifty-four percent of patients had positive blood cultures. In patients with positive blood cultures, the most common causative microorganism was *Escherichia coli* (32.5%), followed by streptococci (19.5%) and staphylococci (16.3%). With regard to the use of antibiotics, carbapenem was most commonly used (55.0%), followed by tazobactam/piperacillin (20.9%) and vancomycin (17.8%) (Table 3).

**Table 1:** Organizational characteristics in the FORECAST study

Characteristics	Number (%) of ICUs	Number (%) of patients
	59	1184
Facility		
Type of ICU		
Open	36 (61.0)	594 (50.2)
Closed	23 (39.0)	590 (49.8)
ICU specialty		
Medical	3 (5.1)	78 (6.6)
Mixed	56 (94.9)	1106 (93.4)
Number of ICU beds		
1–10	25 (42.4)	363 (30.7)
11–20	26 (44.1)	580 (50.0)
≥ 21	8 (13.6)	241 (20.4)
Number of hospital beds		
≤ 500	13 (22.0)	185 (15.6)
501–900	31 (52.5)	637 (53.8)
≥ 901	15 (25.4)	362 (30.6)
Type of hospital		
Non-university	30 (50.8)	458 (38.7)
University	29 (49.2)	726 (61.3)
Staff		
Number of board-certificated intensivists		
0	14 (23.7)	211 (17.8)
1	16 (27.1)	243 (20.5)
≥ 2	29 (49.2)	730 (61.7)
Number of board certificated emergency physicians		
0–4	17 (28.8)	167 (14.1)
5–8	22 (37.3)	321 (27.1)
≥ 9	20 (33.9)	696 (58.8)
Number of ICU nurses		
≤ 40	29 (49.2)	584 (49.3)
> 40	30 (50.8)	600 (50.7)
Nurse to bed ratio in each shift		
1:2	50 (84.7)	1052 (88.9)
1:4	9 (15.3)	132 (11.1)
Patients		
Number of emergency patients (2014) (*n* = 50)		
≤ 4000	14 (28.0))	293 (28.8)
4001–18,000	23 (46.0)	509 (50.0)
≥18,001	13 (26.0)	216 (21.2)
Number of ambulance patients (2014) (*n* = 50)		
≤ 2000	15 (30.0)	313 (30.7)
2001–5000	20 (40.0)	295 (29.0)
≥ 5001	15 (30.0)	410 (40.3)
Number of ICU patients (2013) (*n* = 58)		
≤ 500	15 (25.9)	216 (18.2)
501–1000	28 (48.3)	642 (54.2)
≥ 1001	15 (25.9)	326 (27.5)

*ICU* intensive care unit

**Table 2:** Demographic, infection, and admission characteristics of patients with severe sepsis (*n* = 1184)

Characteristics	
Age at admission, years	73 (64–81)
Male	719/1184 (60.7)
BMI, kg/m^2^	22 (19–25)
Admission source	
ED	676 (57.2)
non-ED (hospital/department transfers)	457 (38.7)
ICU	49 (4.1)
Coexisting conditions	
Myocardial infarction	58 (4.9)
Congestive heart failure	128 (10.8)
Peripheral vascular disease	29 (2.4)
Cerebrovascular disease	140 (11.8)
Dementia	97 (8.2)
COPD	82 (6.9)
Connective tissue disease	83 (7.0)
Peptic ulcer disease	32 (2.7)
Diabetes mellitus without organ damage	197 (16.6)
Diabetes mellitus with organ damage	75 (6.3)
Chronic kidney disease	85 (7.2)
Hemiplegia	44 (3.7)
Malignancy (solid)	161 (13.6)
Malignancy (blood)	23 (1.9)
Metastatic tumor	26 (2.2)
Mild liver disease	46 (3.9)
Moderate to severe liver disease	26 (2.2)
AIDS	1 (0.1)
CCI	
0	391 (33.0)
1–2	542 (45.8)
3–4	182 (15.4)
> 4	69 (5.8)
ADL	
Dependent	288 (24.3)
Suspected site of infection	
Lung	367 (31.0)
Abdomen	311 (26.3)
Urinary tract	218 (18.4)
Soft tissue	117 (9.9)
Central nervous system	23 (1.9)
IV catheter	22 (1.9)
Osteoarticular	21 (1.8)
Endocardium	16 (1.4)
Wound	12 (1.0)
Implant device	8 (0.7)
Other	69 (5.8)
Positive blood cultures	636 (54.0)
Septic shock	
Yes	745 (62.9)
Organ dysfunction on arrival	
Hypotension	656 (55.4)
Hyperlactatemia (> 2 mmol/L)	797 (67.3)
Acute kidney injury (Cre > 2 mg/dL)	455 (38.4)
Acute lung injury	442 (37.3)
Hyperbilirubinemia (> 2.0 mg/dL)	204 (17.2)
Thrombocytopenia (< 100,000/μL)	345 (29.1)
Coagulopathy (INR > 1.5)	225 (19.0)
ARDS^a^ at 1st day	193 (18.0)
qSOFA score	2 (1–3)
qSOFA ≥ 2	800 (69.6)
APACHE II score	23 (17–29)
SIRS score	3 (2–4)
SOFA score	9 (6–11)
SOFA score ≥ 2	987 (98.6)

Reported counts (proportions) for categorical variables and median (interquartile range) for continuous variables

Missing data: body mass index (BMI) = 26, admission source = 2, insurance = 2, activities of daily living (ADL) = 2, blood culture = 7, acute respiratory distress syndrome (ARDS) = 112, quick Sepsis-related Organ Failure Assessment (qSOFA) = 35, Acute Physiology and Chronic Health Evaluation (APACHE) II = 162, systemic inflammatory response syndrome (SIRS) = 43, SOFA = 183

*ED* emergency department, *CCI* Charlson Comorbidity Index, *COPD* chronic obstructive pulmonary disease, *AIDS* acquired immune deficiency syndrome, *IV* intravenous

^a^ARDS was defined by Berlin criteria

**Table 3:** Microbiologic blood culture results and initial antibiotics use among patients with severe sepsis

Microbiological results of blood cultures	*n* = 560
Gram-negative	
*E. coli*	182 (32.5)
Klebsiella	72 (12.9)
Pseudomonas	16 (2.9)
Gram-positive	
Staphylococci	91 (16.3)
Streptococci	109 (19.5)
MRSA	16 (2.9)
Enterococcus	25 (4.5)
Anaerobic	24 (4.3)
Fungi	14 (2.5)
Antibiotics	*n* = 1140
Penicillin derivative (PCG, ABPC, ABPC/MCIPC)	30 (2.6)
Ampicillin/sulbactam	78 (6.8)
PIPC/TAZ	238 (20.9)
Sulbactam/cefoperazone	11 (1.0)
First-generation cephalosporin	28 (2.5)
Second-generation cephalosporin (CTM, CMZ, FMOX)	32 (2.8)
Third-generation cephalosporin (CTX, CPZ, CTRX)	99 (8.7)
Third-generation cephalosporin against pseudomonas	4 (0.4)
Fourth generation cephalosporin against pseudomonas	26 (2.3)
Carbapenem	627 (55.0)
Aminoglycoside	8 (0.7)
Quinolone	19 (1.7)
Tetracycline	7 (0.6)
Macrolide	–
Metronidazole	12 (1.1)
CLDM	48 (4.2)
Vancomycin	203 (17.8)
Other anti-methicillin-resistant *Staphylococcus aureus* drugs	67 (5.9)
Antifungals	50 (4.4)
Others	83 (7.3)

Reported counts (proportions). Missing data: microbiology data = 76, antibiotics = 44. There was no intravenous macrolide during the study period in Japan

*PCG* penicillin G, *ABPC* ampicillin, *ABPC/MCIPC* ampicillin/cloxacillin, *PIPC/TAZ* tazobactam/piperacillin, *CTM* cefotiam, *CMZ* cefmetazole, *FMOX* flomoxef, *CTX* cefotaxime, *CPZ* cefoperazone, *CTRX* ceftriaxone, *CLDM* clindamycin, *CNS* central nervous system

The compliance rate for the entire 3-h bundle was 64.3% (Table 4). Moreover, the rate for each item in the 3-h bundle was high: 96.9% for obtaining serum lactate levels and 76.3% for fluid resuscitation. The compliance rate for the entire 6-h bundles was substantially lower at 3.5%. The compliance rates for vasopressor use and repeat lactate measurement were 88.6% and 90.0%, respectively. However, the compliance rate for the 3-h bundle plus vasopressor use and repeat lactate measurement was 57.3%. A multilevel logistic regression model showed that closed ICUs and non-university hospitals were more compliant with the entire 3-h bundle (Table 5).

**Table 4:** Achievement of Surviving Sepsis Campaign bundle targets in 59 ICUs, Japan (*n* = 1184)

	Compliance rate
Compliance with all applicable elements of sepsis 3-h bundle	
Entire 3-h resuscitation bundle^a^	543/844 (64.3)
B1. Serum lactate obtained	1143/1180 (96.9)
B2. Broad-spectrum antibiotic given	985/1179 (83.5)
B3. Blood cultures obtained before broad-spectrum antibiotic administration	1084/1178 (92.0)
B4. 30 mg/kg crystalloid fluid bolus delivered (yes/cases with indication)^a^	645/845 (76.3)
Compliance with all applicable elements of sepsis 6-h bundle	
Entire 6-h resuscitation bundle^b^	41/688 (3.5)
Vasopressors use + re-measured lactate	625/752 (83.1)
B5. Vasopressor use followed initial fluid bolus if needed to maintain MAP ≥ 65 mmHg (yes/cases with indication)	745/841 (88.6)
B6. CVP measured (yes/cases with indication)^a^	225/856 (26.3)
B7. CVP 8 mmHg achieved (yes/cases with indication)^a^	181/227 (79.7)
B8. ScvO2 measured (yes/cases with indication)^a^	60/853 (7.0)
B9. ScvO2 70% (or SvO2 65%) achieved (yes/cases with indication)^a^	49/66 (74.2)
B10. Re-measured lactate if initial lactate elevated (yes/cases with indication)	864/960 (90.0)
Entire 3-h resuscitation bundle + vasopressors use + re-measured lactate	397/693 (57.3)
Entire 3-h resuscitation bundle + vasopressors use	449/750 (59.9)

Reported counts (proportions). Missing data: B1 = 4, B2 = 5, B3 = 6, B4 = 9, B5 = 7, B6 = 10, B7 = 757, B8 = 9, B9 = 899, B10 = 14

*MAP* mean arterial pressure, *CVP* central venous pressure, *ScvO2* central venous oxygen saturation, *SvO2* mixed venous oxygen saturation, *ICU* intensive care unit

^a^Septic shock or lactate > 4 mmol/L

^b^Excluded the achievement of a CVP of 8 mmHg and a ScvO2 of 70% from the entire 6-h bundle analysis

**Table 5:** A multilevel logistic model for achievement of the entire 3-h resuscitation bundle among ICUs in the FORECAST study

Characteristics	Odds_(95%CI)
Type of ICU	
Closed	2.84_(1.28–6.28)
Number of ICU beds	
1–10	
11 to 20	0.88_(0.38–2.08)
≥ 21	1.57_(0.44–5.6)
Type of hospital	
University	0.35_(0.15–0.78)
Number of board-certified intensivists	
0	
1	0.7_(0.25–1.95)
≥ 2	0.62_(0.24–1.64)
ICU nurse ratio	
1:2	1.24_(0.41–3.75)
Number of ICU patients per year	
0–500	
501–1000	1.22_(0.48–3.14)
≥ 1001	2.18_(0.61–7.78)

*ICU* intensive care unit

**Fig. 1:**
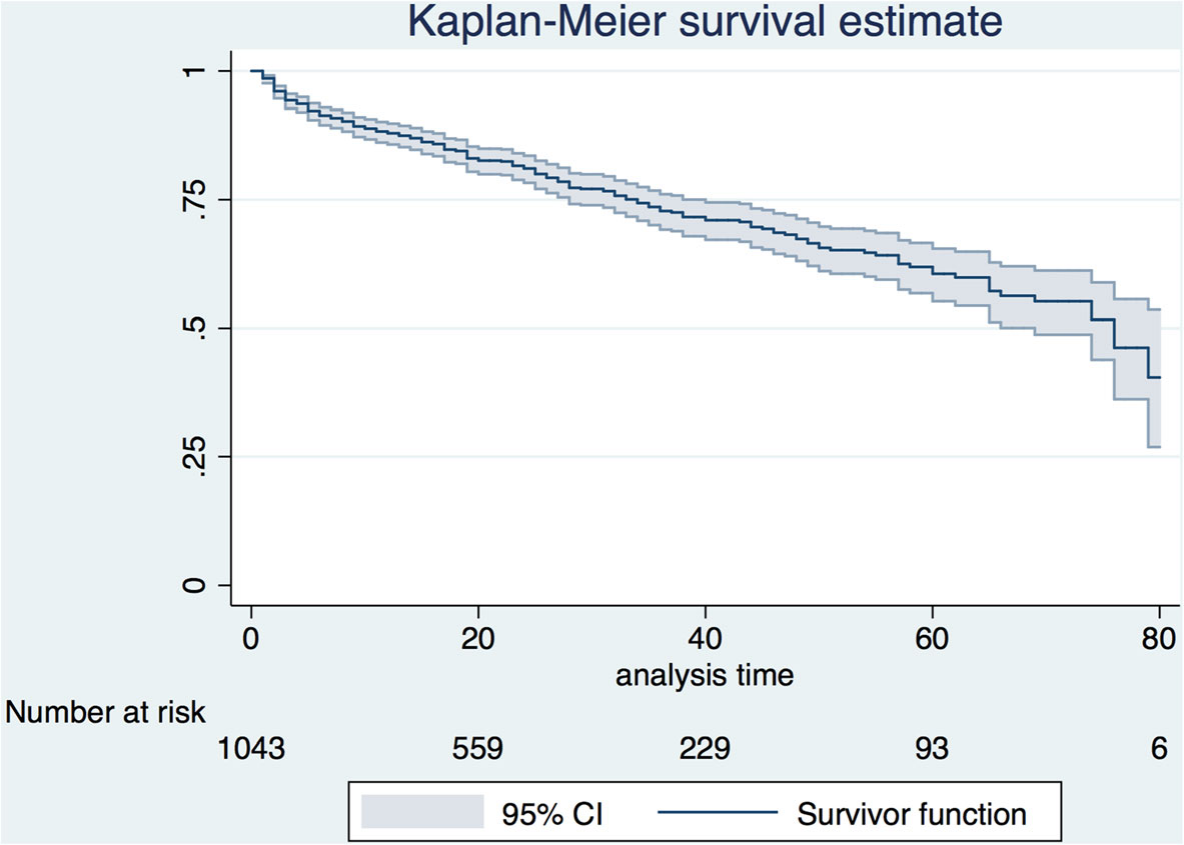
Survival probability during the first 80 days for patients with severe sepsis (*n* = 1043)

The overall in-hospital mortality rate was 23.4% (21.0–26.0) (Table 6). The mortality rate in patients with septic shock was 27.9% (24.6–31.3). However, the in-hospital mortality rate in patients with non-septic shock was 16.0% (12.7–19.9). Among the survivors, 36.7% of patients were discharged home. The median number of ICU-free days was 19 (IQR 11–24). The median number of VFD was 21 (IQR 0–28), and the LOS was 24 (12–46) days. The mortality rate continued to increase even after the acute phase of severe sepsis (Fig. 1). Older age, multiple comorbidities (CCI), suspected site of infection (the condition of patients with intra-abdominal or urinary tract infection was less severe than that of patients with pneumonia), and increasing SOFA scores correlated with in-hospital mortality based on the GEE model. However, gender, BMI, ADL, admission source, presence of shock, bacteremia, utilization of the fluid resuscitation protocol, and use of broad-spectrum antibiotics were not correlated with in-hospital mortality in the model (Table 7).

**Table 6:** Outcomes among patients with severe sepsis (*n* = 1184)

Outcomes	
In-hospital mortality	269/1148 (23.4 (21.0–26.0)%)
with shock (*n* = 745)	200/718 (27.9 (24.6–31.3)%)
Survivor disposition	
Home	323/879 (36.7)
Transfer	556/879 (63.3)
ICU-free days	19 (11–24)
Ventilator-free days	21 (0–28)
Length of hospital stay	24 (12–46)

Reported counts (proportions) for categorical and median (interquartile range) for continuous variables

Missing data: in-hospital mortality=36, ICU free days=263, Ventilator free days=47

*ICU* intensive care unit

**Table 7:** Relationship between epidemiological and treatment factors and in-hospital mortality among patients with severe sepsis using a generalized estimating equation model (*n* = 952)

Characteristics	Odds_(95%CI)
Age at admission, years	1.02_(1.01–1.04)
Male sex	0.81_(0.57–1.14)
Body mass index	1_(0.97–1.04)
ADL	0.88_(0.58–1.32)
Admission source	
ED	reference
w/o ED (transfer or other department)	1.12_(0.78–1.62)
ICU	1.99_(0.91–4.36)
CCI	1.2_(1.09–1.32)
Septic shock	
Yes	1.01_(0.63–1.62)
Suspected site of infection	
Lung	reference
Abdomen	0.54_(0.35–0.85)
Urinary tract	0.22_(0.12–0.41)
Soft tissue	0.73_(0.4–1.33)
Others	1.16_(0.7–1.89)
SOFA score	1.18_(1.12–1.24)
Positivity of blood cultures (bacteremia)	1.16_(0.82–1.64)
Fluid resuscitation protocol^a^	
Not achievement	reference
Achievement	1.14_(0.72–1.82)
Not indicated	1.06_(0.59–1.91)
Broad-spectrum antibiotic use	1.04_(0.66–1.64)

Missing data: body mass index = 26, admission source = 2, ADL = 2, positivity of blood cultures = 7, Sepsis-related Organ Failure Assessment (SOFA) = 183, fluid resuscitation protocol = 9, broad-spectrum antibiotic use = 5

*ED* emergency department, *ICU* intensive care unit, *CCI* Charlson Comorbidity Index, *ADL* activities of daily living

^a^Crystalloid fluid bolus (30 mg/kg) delivered if it was indicated in the bundle in Surviving Sepsis Campaign Guidelines 2012

## Discussion

### Summary

In this study, we described the epidemiology and outcomes associated with severe sepsis in Japan to better understand how clinicians adhere to guidelines in routine clinical practice. Our study population included elderly individuals with various comorbidities, and this is an accurate reflection of the super-aging society in Japan. The in-hospital mortality rate in our study in Japan was comparable to that of other developed countries. Sepsis management in our study was characterized by high compliance with 3-h bundles and low compliance with CVP and ScvO2 measurements. The mortality rate continued to increase even after the acute phase of severe sepsis, although one third of the survivors were discharged home after recovery.

Observational (non-interventional) studies on severe sepsis have been recently decreasing because of its changing definition. The age of study populations around the world has been rapidly increasing in prospective multicenter cohort studies among patients with severe sepsis (Additional file 1: Table S1). Additional file 1 shows a summary of these studies. As an Asian comparison, a nationwide epidemiological study on sepsis in Taiwan, analyzed a similar aging cohort with sepsis [10], and their results showed that diabetes mellitus (with or without complications) was the most common comorbidity, which is consistent with that of our study. This likely reflects the common characteristics of individuals in developed countries, although it remains controversial whether the severity of diabetes mellitus itself is a risk factor for sepsis [11, 12]. Previous studies have shown that aging and multiple comorbidities were the risk factors associated with poor outcome due to sepsis [1, 7, 13–16]; however, a single comorbidity was not. The distribution of suspected infection sites was similar to that in previous reports [13, 17]. This similarity suggests that our results showing the effect of site of infection on outcomes may be generalizable to other countries [13, 17].

In our study, blood culture tests were obtained in most cases, and half of the patients were positive for blood culture tests. Although it remains unclear whether bacteremia itself correlate with mortality in individuals with sepsis [13, 16, 18], we believe that the relatively high prevalence of bacteremia likely correlates with severity, particularly considering the frequent occurrence of septic shock. Otherwise, it suggests a higher bacterial load or utilization of a more sensitive technique, although information about the blood culture technique is not available. In the FORECAST study, we identified septic shock based on blood pressure measurements and vasopressor requirements, whereas clinical diagnosis was used in our previous registry [5]. Though the definition of septic shock has not changed between these studies, the prevalence of septic shock was significantly higher in this study than that in the previous study, which is irrespective of similar severities identified using the SOFA and APACHE II scores or mortality. Presumably, early recognition of septic shock has persisted after the introduction of the SSC guidelines [8, 19].

Our current study showed a high compliance rate for the entire 3-h bundle, but a low compliance rate for the entire 6-h bundle. This discrepancy may have been influenced by recent studies, such as those showing that high compliance with the 3-h bundle is associated with lower in-hospital mortality [20]. In our study, the low compliance rate for the entire 6-h bundle was significantly influenced by the lack of central venous catheter (CVC) measurements. This may be due to the fact that CVP measurement has recently been considered as a suboptimal guide for fluid resuscitation in circulatory shock [21]. Both systematic reviews [22] and guidelines [19] suggest that CVP values alone should not be used to determine the prognosis of patients with septic shock. The compliance rate of lactate measurement was high in our study, so lactate measurement may be used as a surrogate strategy for ScvO2 assessment in Japan, as some guidelines weakly recommend this [9, 19]. Since the SSCG has not been recommending the use of CVC to monitor CVP and ScvO2 in patients with septic shock since 2015 after obtaining the results of three trials [23–25], the use of lactate levels as a marker of tissue hypoperfusion may be more reasonable, in addition to the fact that is easier to use in clinical settings. These results highlight the importance of the 1-h bundle [9] and may prevent the need for the 6-h bundle, although some of its elements may remain.

Our previous observational study showed that the in-hospital mortality of patients with severe sepsis correlated with compliance with the bundles, despite the low compliance rates [26]. A global observational study in 2013 had supported this association [27]. Regarding initial resuscitation, bundle compliance in the current study improved substantially [26, 27]. This may be circumstantial evidence about the awareness of and education on sepsis, as well as the achievement of adequate implementation of evidence-based guidelines [28]. This result might have led to similar discussions about resuscitation in individuals with septic shock using the early goal direct therapy (EGDT) [23, 24]. Moreover, 3-h bundles may have simply increased the utilization of ICU resources like the routine implementation of EGDT [29]. Indeed, the bundles have become the cornerstone of improving the quality of sepsis management, as stated in the updated 2018 guidelines [9]. Our compliance with the 3-h bundles plus vasopressor use and repeat lactate measurement was a proper surrogate marker of the updated sepsis bundle [9]; however, greater effort must be applied to consistently achieve this within 1 h (1-h bundle).

The institutional and individual background on studies that complied with sepsis bundles and factors related to reduced mortality are varied [30]. Moreover, a previous study on the compliance with sepsis bundles in Asia [31], a sub-study in Korea from the same database [32], and our results have shown different factors related to favorable outcomes. Guideline compliance is always influenced by the social economic status of each country. In fact, sufficient knowledge and attitude and elimination of external barriers would help improve compliance with the bundles [33]. Performance improvement programs and quality improvement initiatives (rather than types of institution) would be useful to overcome each country’s social and economic problems and to improve the quality of sepsis management [30].

Regarding the outcomes, a quarter of patients with severe sepsis died during hospitalization. Patients with severe sepsis were intubated and admitted to the ICUs for approximately 1 week. They were admitted to the hospital for 3 weeks on average. The in-hospital mortality rate in patients with septic shock was approximately 47% according to a systematic review [34]. However, in a more recent meta-analysis of interventional studies comparing EGDT and conventional resuscitation in developed countries, the in-hospital mortality rate of patients with septic shock was substantially lower at 23% [29]. We should be cautious in comparing our results with those of previous interventional studies that excluded complicated cases. The in-hospital mortality rate (ranging from 28.0% to 55.7%) in patients with severe sepsis in multicenter prospective non-interventional cohort studies conducted since 2001 was more comparable to that of the present study (Additional file 1: Table S1). Additional file 1 shows this in more detail. The global and population-level estimate of in-hospital mortality among patients with severe sepsis in high-income countries was approximately 26% in a meta-analysis conducted from 2003 to 2015 [35]. In addition, in developed countries, the mortality rate of patients with severe sepsis has been gradually declining [35], although it is difficult to distinguish whether this trend reflects accurate improvements in prognosis or the more frequent inclusion of mild sepsis. However, the fact that the early recognition of sepsis has improved patient outcomes worldwide is not disputable.

This study showed that the different epidemiological and treatment factors have effects on mortality. Our results confirmed the findings of previous studies showing that older age [7], multiple comorbidities (CCI) [7], site of infection [13], and SOFA score [36] correlate with mortality. However, the relationship between admission source [37], presence of shock or bacteremia [13], and in-hospital mortality in patients with sepsis remains controversial. Patients with shock had a high mortality rate. By contrast, a potentially severe factor may not be shock but a constitutive factor of shock, such as infection site [13, 17, 38]. Although ADL was not correlated with in-hospital mortality in this super-aging population based on our results, future studies must be conducted on conditions significantly associated with age, comorbidities, and frailty. A previous study has shown that being overweight or obese reduces the adjusted mortality rate in patients with sepsis [39, 40]. Because the number of overweight patients in our study was limited, we may focus on underweight patients as the Asian population [41]. Regarding resuscitation and treatment, the use of the fluid resuscitation protocol and broad-spectrum antibiotics did not show favorable results. In fact, education about the implementation of the sepsis bundle has been effective, as evidenced by a high compliance rate. The efficacy of using fluid resuscitation and broad-spectrum antibiotics alone for sepsis care may be limited.

During hospitalization, intubation days and days in the ICU may be better indicators of the prognosis of patients with severe sepsis. By contrast, the overall LOS usually reflects not only disease prognosis but also sociodemographic and system-based factors. For example, a patient may not be able to live alone even after recovery from severe sepsis; indeed, only one third of our patients were discharged home, and the mortality rates continued to increase for 3 months after survival from acute sepsis. This result is not surprising considering the super-aging society of Japan; despite the decreasing in-hospital mortality rates, we expect that the mortality rate after acquiring sepsis will remain high for quite some time [1, 42]. Unfortunately, in this study we did not obtain data after hospitalization, such as data on late mortality and quality of life after sepsis. Whether sepsis itself correlates with late mortality in individuals with sepsis or that the risk factors for sepsis, such as aging and comorbidities, correlate with late mortality remains controversial [1, 43]. Long-term quality of life after developing sepsis is more important regardless of the relationship.

### Limitations

This study has several important limitations that warrant discussion. First, there might be a selection bias because the study only included patients admitted to the ICUs of tertiary-level emergency care facilities, particularly in hospitals of high complexity. A significant number of patients may have been treated outside of the ICU. Second, the 59 ICUs included in the study only comprise one fifth of the total tertiary emergency facilities in Japan, and this may have introduced sampling bias. Nonetheless, the characteristics of the patients were similar to those reported in other studies conducted in Japan and other countries. Third, the descriptive nature of the study could not fundamentally identify the causal relationship between the observed characteristics and outcomes. Fourth, we excluded the CVP and ScvO2 measurement from the evaluation of SSCG bundles due to large numbers of missing data. CVP of 8 mmHg and ScvO2 of 70% may have been alternatives, as these criteria were also the targets of resuscitation at that time. Fifth, information in our database about ICU stay and ventilator days were not included because they could have been underestimated compared with ICU-free days and VFD. Finally, the patients were not followed (e.g., via phone calls) after their discharge from the hospital, and such outcomes after discharge are not properly assessed. However, most patients in Japan usually return to the hospital after a previous admission, providing at least some confidence in assessing long-term prognoses.

## Conclusions

Our prospective study showed that sepsis management in Japan was characterized by high compliance with the 3-h bundle and low compliance with CVC measurement in the 6-h bundle. The in-hospital mortality in our study was comparable to that of other studies in developed countries. Only one third of the patients were discharged home, given the aging population with comorbidities in ICUs in Japan. International standard therapies should be targeted toward elderly populations, and they must be easily utilized. Efforts toward improving outcomes for both patients and survivors by the national or global registries must be further monitored.

## Key messages

- Patients with severe sepsis in Japan were super-elderly with comorbidities, with in-hospital mortality comparable to other developed countries.
- Sepsis management in Japan was characterized by a high compliance with the 3-h bundle and low compliance with the CVC measurement in the 6-h bundle.
- Mortality continued to increase even after an acute phase of severe sepsis, although one third of survivors were discharged to their homes after recovery.

## Additional files

Additional file 1: **Table S1.** Prospective multicenter cohort studies (noninterventional) among patients with severe sepsis since 2001. The results from a systematic review of prospective multicenter cohort studies (noninterventional) among patients with severe sepsis since 2001. (DOCX 19 kb)

Additional file 2: FORECAST steering committee. The member list of FORECAST steering committee. (DOCX 15 kb)

## Abbreviations

ADL: Activities of daily living
APACHE II: Acute Physiologic Assessment and Chronic Health Evaluation II
BMI: Body mass index
CCI: Charlson comorbidity index
CVP: Central venous pressure
ED: Emergency department
EGDT: Early goal direct therapy
FIO2: Fraction of inspired oxygen
FORECAST: Focused Outcomes Research in Emergency Care in Acute Respiratory Distress Syndrome, Sepsis, and Trauma
GEE: Generalized estimating equation
ICUs: Intensive care units
IQR: Interquartile range
LOS: Length of hospital stay
MAP: Mean arterial pressure
PaO2: Partial pressure of arterial oxygen
ScvO2: Central venous oxygen saturation
SOFA: Sepsis-related Organ Failure Assessment
SSC: Surviving Sepsis Campaign
SSCG: Surviving Sepsis Campaign Guidelines
UMIN-CTR: University Hospital Medical Information Network Clinical Trials Registry
VFD: Ventilator-free days

## References

[1] Prescott HC, Osterholzer JJ, Langa KM, Angus DC, Iwashyna TJ (2016). Late mortality after sepsis: propensity matched cohort study. BMJ.

[2] Angus DC, Linde-Zwirble WT, Lidicker J, Clermont G, Carcillo J, Pinsky MR (2001). Epidemiology of severe sepsis in the United States: analysis of incidence, outcome, and associated costs of care. Crit Care Med.

[3] Slade E, Tamber PS, Vincent JL (2003). The Surviving Sepsis Campaign: raising awareness to reduce mortality. Crit Care.

[4] Reinhart K, Daniels R, Kissoon N, Machado FR, Schachter RD, Finfer S (2017). Recognizing Sepsis as a global health priority - a WHO resolution. N Engl J Med.

[5] Ogura H, Gando S, Saitoh D, Takeyama N, Kushimoto S, Fujishima S, Mayumi T, Araki T, Ikeda H, Kotani J (2014). Epidemiology of severe sepsis in Japanese intensive care units: a prospective multicenter study. J Infect Chemother.

[6] Levy MM, Fink MP, Marshall JC, Abraham E, Angus D, Cook D, Cohen J, Opal SM, Vincent JL, Ramsay G (2003). 2001 SCCM/ESICM/ACCP/ATS/SIS International Sepsis Definitions Conference. Crit Care Med.

[7] Yang Y, Yang KS, Hsann YM, Lim V, Ong BC (2010). The effect of comorbidity and age on hospital mortality and length of stay in patients with sepsis. J Crit Care.

[8] Dellinger RP, Levy MM, Rhodes A, Annane D, Gerlach H, Opal SM, Sevransky JE, Sprung CL, Douglas IS, Jaeschke R (2013). Surviving Sepsis Campaign: international guidelines for management of severe sepsis and septic shock: 2012. Crit Care Med.

[9] Levy MM, Evans LE, Rhodes A (2018). The Surviving Sepsis Campaign bundle: 2018 update. Intensive Care Med.

[10] Lee CC, Yo CH, Lee MG, Tsai KC, Lee SH, Chen YS, Lee WC, Hsu TC, Lee SH, Chang SS (2017). Adult sepsis - a nationwide study of trends and outcomes in a population of 23 million people. J Inf Secur.

[11] Lepper PM, Ott S, Nuesch E, von Eynatten M, Schumann C, Pletz MW, Mealing NM, Welte T, Bauer TT, Suttorp N (2012). Serum glucose levels for predicting death in patients admitted to hospital for community acquired pneumonia: prospective cohort study. BMJ.

[12] Joshi N, Caputo GM, Weitekamp MR, Karchmer AW (1999). Infections in patients with diabetes mellitus. N Engl J Med.

[13] Jeganathan N, Yau S, Ahuja N, Otu D, Stein B, Fogg L, Balk R (2017). The characteristics and impact of source of infection on sepsis-related ICU outcomes. J Crit Care.

[14] Martin GS, Mannino DM, Moss M (2006). The effect of age on the development and outcome of adult sepsis*. Crit Care Med.

[15] Blot S, Cankurtaran M, Petrovic M, Vandijck D, Lizy C, Decruyenaere J, Danneels C, Vandewoude K, Piette A, Vershraegen G (2009). Epidemiology and outcome of nosocomial bloodstream infection in elderly critically ill patients: a comparison between middle-aged, old, and very old patients. Crit Care Med.

[16] Vincent J-L, Sakr Y, Sprung CL, Ranieri VM, Reinhart K, Gerlach H, Moreno R, Carlet J, Le Gall J-R, Payen D (2006). Sepsis in European intensive care units: results of the SOAP study*. Crit Care Med.

[17] Leligdowicz A, Dodek PM, Norena M, Wong H, Kumar A, Kumar A, Co-operative Antimicrobial Therapy of Septic Shock Database Research Group (2014). Association between source of infection and hospital mortality in patients who have septic shock. Am J Respir Crit Care Med.

[18] Kumar A, Roberts D, Wood KE, Light B, Parrillo JE, Sharma S, Suppes R, Feinstein D, Zanotti S, Taiberg L (2006). Duration of hypotension before initiation of effective antimicrobial therapy is the critical determinant of survival in human septic shock. Crit Care Med.

[19] Rhodes A, Evans LE, Alhazzani W, Levy MM, Antonelli M, Ferrer R, Kumar A, Sevransky JE, Sprung CL, Nunnally ME (2017). Surviving Sepsis Campaign: International guidelines for management of sepsis and septic shock: 2016. Crit Care Med.

[20] Seymour CW, Gesten F, Prescott HC, Friedrich ME, Iwashyna TJ, Phillips GS, Lemeshow S, Osborn T, Terry KM, Levy MM (2017). Time to treatment and mortality during mandated emergency care for sepsis. N Engl J Med.

[21] Cecconi M, De Backer D, Antonelli M, Beale R, Bakker J, Hofer C, Jaeschke R, Mebazaa A, Pinsky MR, Teboul JL (2014). Consensus on circulatory shock and hemodynamic monitoring. Task force of the European Society of Intensive Care Medicine. Intensive Care Med.

[22] Eskesen TG, Wetterslev M, Perner A (2016). Systematic review including re-analyses of 1148 individual data sets of central venous pressure as a predictor of fluid responsiveness. Intensive Care Med.

[23] Pro CI, Yealy DM, Kellum JA, Huang DT, Barnato AE, Weissfeld LA, Pike F, Terndrup T, Wang HE, Hou PC (2014). A randomized trial of protocol-based care for early septic shock. N Engl J Med.

[24] Investigators A, Peake SL, Delaney A, Bailey M, Bellomo R, Cameron PA, Cooper DJ, Higgins AM, Holdgate A, Group ACT (2014). Goal-directed resuscitation for patients with early septic shock. N Engl J Med.

[25] Mouncey PR, Osborn TM, Power GS, Harrison DA, Sadique MZ, Grieve RD, Jahan R, Harvey SE, Bell D, Bion JF (2015). Trial of early, goal-directed resuscitation for septic shock. N Engl J Med.

[26] Fujishima S, Gando S, Saitoh D, Mayumi T, Kushimoto S, Shiraishi S, Ogura H, Takuma K, Kotani J, Ikeda H (2014). A multicenter, prospective evaluation of quality of care and mortality in Japan based on the Surviving Sepsis Campaign guidelines. J Infect Chemother.

[27] Rhodes A, Phillips G, Beale R, Cecconi M, Chiche JD, De Backer D, Divatia J, Du B, Evans L, Ferrer R (2015). The Surviving Sepsis Campaign bundles and outcome: results from the International Multicentre Prevalence Study on Sepsis (the IMPreSS study). Intensive Care Med.

[28] Liu VX, Morehouse JW, Marelich GP, Soule J, Russell T, Skeath M, Adams C, Escobar GJ, Whippy A (2016). Multicenter implementation of a treatment bundle for patients with sepsis and intermediate lactate values. Am J Respir Crit Care Med.

[29] Angus DC, Barnato AE, Bell D, Bellomo R, Chong CR, Coats TJ, Davies A, Delaney A, Harrison DA, Holdgate A (2015). A systematic review and meta-analysis of early goal-directed therapy for septic shock: the ARISE, ProCESS and ProMISe Investigators. Intensive Care Med.

[30] Damiani E, Donati A, Serafini G, Rinaldi L, Adrario E, Pelaia P, Busani S, Girardis M (2015). Effect of performance improvement programs on compliance with sepsis bundles and mortality: a systematic review and meta-analysis of observational studies. PLoS One.

[31] Phua J, Koh Y, Du B, Tang YQ, Divatia JV, Tan CC, Gomersall CD, Faruq MO, Shrestha BR, Gia Binh N (2011). Management of severe sepsis in patients admitted to Asian intensive care units: prospective cohort study. BMJ.

[32] Kim JH, Hong SK, Kim KC, Lee MG, Lee KM, Jung SS, Choi HS, Lee JH, Jung KS, Lee SS (2012). Influence of full-time intensivist and the nurse-to-patient ratio on the implementation of severe sepsis bundles in Korean intensive care units. J Crit Care.

[33] Cabana MD, Rand CS, Powe NR, Wu AW, Wilson MH, Abboud PA, Rubin HR (1999). Why don't physicians follow clinical practice guidelines? A framework for improvement. JAMA.

[34] Shankar-Hari M, Phillips GS, Levy ML, Seymour CW, Liu VX, Deutschman CS, Angus DC, Rubenfeld GD, Singer M, Sepsis Definitions Task F (2016). Developing a new definition and assessing new clinical criteria for septic shock: for the Third International Consensus Definitions for Sepsis and Septic Shock (Sepsis-3). JAMA.

[35] Fleischmann C, Scherag A, Adhikari NK, Hartog CS, Tsaganos T, Schlattmann P, Angus DC, Reinhart K, International Forum of Acute Care T (2016). Assessment of global incidence and mortality of hospital-treated sepsis. current estimates and limitations. Am J Respir Crit Care Med.

[36] Seymour CW, Liu VX, Iwashyna TJ, Brunkhorst FM, Rea TD, Scherag A, Rubenfeld G, Kahn JM, Shankar-Hari M, Singer M (2016). Assessment of clinical criteria for sepsis: for the Third International Consensus Definitions for Sepsis and Septic Shock (Sepsis-3). JAMA.

[37] van Vught LA, Klein Klouwenberg PM, Spitoni C, Scicluna BP, Wiewel MA, Horn J, Schultz MJ, Nurnberg P, Bonten MJ, Cremer OL (2016). Incidence, risk factors, and attributable mortality of secondary infections in the intensive care unit after admission for sepsis. JAMA.

[38] Kushimoto S, Gando S, Ogura H, Umemura Y, Saitoh D, Mayumi T, Fujishima S, Abe T, Shiraishi A, Ikeda H, et al. Complementary role of hypothermia identification to the quick Sequential Organ Failure Assessment score in predicting patients with sepsis at high risk of mortality: a retrospective analysis from a multicenter, observational study. J Intensive Care Med. 2018:885066618761637. PMID: 29544388. [Epub ahead of print]. 10.1177/0885066618761637 29544388

[39] Kuperman EF, Showalter JW, Lehman EB, Leib AE, Kraschnewski JL (2013). The impact of obesity on sepsis mortality: a retrospective review. BMC Infect Dis.

[40] Pepper DJ, Sun J, Welsh J, Cui X, Suffredini AF, Eichacker PQ (2016). Increased body mass index and adjusted mortality in ICU patients with sepsis or septic shock: a systematic review and meta-analysis. Crit Care.

[41] Yamazaki K, Suzuki E, Yorifuji T, Tsuda T, Ohta T, Ishikawa-Takata K, Doi H (2017). Is there an obesity paradox in the Japanese elderly population? A community-based cohort study of 13 280 men and women. Geriatr Gerontol Int.

[42] Iwashyna TJ, Cooke CR, Wunsch H, Kahn JM (2012). Population burden of long-term survivorship after severe sepsis in older Americans. J Am Geriatr Soc.

[43] Shankar-Hari M, Ambler M, Mahalingasivam V, Jones A, Rowan K, Rubenfeld GD (2016). Evidence for a causal link between sepsis and long-term mortality: a systematic review of epidemiologic studies. Crit Care.

