# Supplementary material for: Characteristics, management, and in-hospital mortality among patients with severe sepsis in intensive care units in Japan: the FORECAST study
Source: Crit Care. 2018 Nov 22;22:322. doi: 10.1186/s13054-018-2186-7 (PMC6251147; doi:10.1186/s13054-018-2186-7)
Supplement: Supplementary file 1 — Table S1. Prospective multicenter cohort studies (noninterventional) among patients with severe sepsis since 2001. The results from a systematic review of prospective multicenter cohort studies (noninterventional) among patients with severe sepsis since 2001. (DOCX 19 kb) [file 13054_2018_2186_MOESM1_ESM.docx]

Table S1**:**  Prospective multicenter cohort studies (noninterventional) among patients with severe sepsis since 2001

|  | Year | Setting | Patient number | Age  (y.o.) | APACHE II score | SOFA score | In-hospital mortality (%) | In-hospital mortality (%) [septic shock] |
| --- | --- | --- | --- | --- | --- | --- | --- | --- |
| Brun-Buisson, 2004 | 2001 | France | 546 | 65^a^ | ND | 9 (range 1-24)^a^ | 41.9 | ND |
| Engel, 2007 | 2003 | Germany | 415 | 67^a^ | 19 (IQR 13-24)^a^ | 8 (IQR 5-11)^a^ | 55.2 | ND |
| Cheng, 2007 | 2004-2005 | China | 318 | 64^a^ | 19 (IQR 14-25)^a^ | 8 (IQR 6-12)^a^ | 48.7 | ND |
| Karlsson, 2007 | 2004-2005 | Finland | 470 | 59.6^b^ | 24.1 (± 9.1)^b^ | ND | 28.3 | ND |
| Blanco, 2008 | 2002 | Spain | 311 | 68^a^ | 25.5 (± 7.1)^b^ | 9.6 (± 3.7)^b^ | 54.3 | ND |
| Beale, 2009 | 2002-2005 | 37 countries | 12,881 | 60.4^b^ | 23.3 (± 8.3)^b^ | 9.3 (± 3.9)^b^ | 49.6 | ND |
| Park, 2012 | 2005-2009 | South Korea | 1,192 | 65.0^b^ | 18.8 (± 7.3)^b^ | 7.5 (± 3.9)^b^ | 28.0 | ND |
| Zhou, 2014 | 2009 | China | 484 | 66 ^a^ | 21 (IQR 16-27)^a^ | 7.5 (IQR 5-10)^a^ | 33.5 | ND |
| Ogura, 2014 | 2010-2011 | Japan | 624 | 69.0^b^ | 23.4 (± 8.3)^b^ | 8.6 (± 4.0)^b^ | 29.5 | 41.5 |
| Marx, 2016 | 2013 | Germany | 1503 | 71^a^ | ND | ND (SAPSII 45 ^a^) | 40.4 | 43.3 |
| Baykara, 2018 | 2016 | Turkey | 260 (w/o shock)  203 (w/ shock) | 69^a^  70 ^a^ | 21.5 (IQR17-28)^a^  25 (IQR 19-31)^a^ | 8 (IQR 6-11)^a^  10 (IQR 7-13)^a^ | 55.7 | 70.4 |
| Present study | 2016-2017 | Japan | 1184 | 73^a^ | 23 (IQR 17-29)^a^ | 9 (IQR 6-11)^a^ | 23.4 | 27.9 |
| ND=no data, IQR=interquartile, APACHE=acute physiology and chronic health evaluation, SOFA=sequential organ failure assessment ^a^median, ^b^mean (±standard deviation), w/o:without, w/:with | | | | | | | | |
