# Supplementary material for: Characteristics, management, and in-hospital mortality among patients with severe sepsis in intensive care units in Japan: the FORECAST study
Source: Crit Care. 2018 Nov 22;22:322. doi: 10.1186/s13054-018-2186-7 (PMC6251147; doi:10.1186/s13054-018-2186-7)
Supplement: Supplementary file 2 — FORECAST steering committee. The member list of FORECAST steering committee. (DOCX 15 kb) [file 13054_2018_2186_MOESM2_ESM.docx]

**FORECAST steering committee**

1. Department of General Medicine, Juntendo University, Japan (Toshikazu Abe)
2. Department of Traumatology and Acute Critical Medicine, Osaka University Graduate School of Medicine, Japan (Hiroshi Ogura, Yutaka Umemura)
3. Emergency and Trauma Center, Kameda Medical Center, Japan (Atsushi Shiraishi)
4. Division of Emergency and Critical Care Medicine, Tohoku University Graduate School of Medicine, Japan (Shigeki Kushimoto)
5. Division of Traumatology, Research Institute, National Defense Medical College (Daizoh Saitoh)
6. Center for General Medicine Education, Keio University School of Medicine, Japan (Seitaro Fujishima)
7. Department of Emergency Medicine, School of Medicine, University of Occupational and Environmental Health, Japan (Toshihiko Mayumi)
8. Department of Acute Medicine, Kawasaki Medical School, Japan (Yasukazu Shiino)
9. Department of Emergency and Critical Care Medicine Chiba University Graduate School of Medicine, Japan (Taka-aki Nakada)
10. Department of Trauma and Critical Care Medicine, Kyorin University School of Medicine, Japan (Takehiko Tarui)
11. Department of Emergency and Critical Care Medicine, St. Luke's International Hospital, Japan (Toru Hifumi)
12. Trauma and Acute Critical Care Center, Medical Hospital, Tokyo Medical and Dental University, Japan (Yasuhiro Otomo)
13. Department of Surgery, Center for Gastroenterology and Liver Disease, Kitakyushu City Yahata Hospital, Japan (Kohji Okamoto)
14. Department of Disaster and Emergency Medicine, Kobe University Graduate School of Medicine, Japan (Joji Kotani)
15. Emergency and Critical Care Medicine, Saga University Hospital, Japan (Yuichiro Sakamoto)
16. Department of Emergency and Critical Care Medicine, Keio University School of Medicine, Japan (Junichi Sasaki)
17. Department of Emergency and Critical Care Medicine, Aizu Chuo Hospital, Japan (Shin-ichiro Shiraishi)
18. Emergency & Critical Care Center, Kawasaki Municipal Kawasaki Hospital, Japan (Kiyotsugu Takuma)
19. Advanced Medical Emergency & Critical Care Center, Yamaguchi University Hospital, Japan (Ryosuke Tsuruta)
20. Center Hospital of the National Center for Global Health and Medicine, Japan (Akiyoshi Hagiwara)
21. Division of Trauma and Surgical Critical Care, Osaka General Medical Center, Japan (Kazuma Yamakawa)
22. Department of Emergency and Critical Care Medicine, Nippon Medical School, Japan (Tomohiko Masuno)
23. Advanced Critical Care Center, Aichi Medical University Hospital, Japan (Naoshi Takeyama)
24. Advanced Emergency Medical Service Center Kurume University Hospital, Japan (Norio Yamashita)
25. Department of Emergency Medicine, Teikyo University School of Medicine, Japan (Hiroto Ikeda)
26. Department of Trauma, Critical Care Medicine, and Burn Center, Japan Community Healthcare Organization, Chukyo Hospital, Japan (Masashi Ueyama)
27. Division of Acute and Critical Care Medicine, Hokkaido University Graduate School of Medicine, Japan (Satoshi Gando)
